# Incidence and characteristics of prehospital fatalities from haemorrhage in Sweden: a nationwide observational study

**DOI:** 10.1186/s13049-024-01269-z

**Published:** 2024-09-26

**Authors:** Oliver von Olnhausen, Andreas Wladis, Denise Bäckström

**Affiliations:** 1 Perioperative Medicine and Intensive Care, Karolinska University Hospital, Stockholm, Sweden; 2 Department of Biomedical and Clinical Sciences, Linköping University, Linköping, Sweden

**Keywords:** Haemorrhage, Prehospital, Haemorrhagic shock, Massive bleeding, Trauma

## Abstract

**Background:**

Haemorrhage is a leading cause of preventable mortality in high-income countries and emergency management presents unique challenges in the prehospital setting. The study aimed to determine incidence and characteristics of fatalities from prehospital haemorrhage in Sweden.

**Methods:**

A nationwide retrospective cohort study 2012–2021 was conducted using data from the Swedish National Board of Health and Welfare. Prehospital fatality from haemorrhage was defined as a cause of death related to haemorrhage (Appendix 1) without a hospital admission on the same day. Primary outcome was age-standardized mortality rate per 100,000 inhabitants.

**Results:**

A total of 9801 prehospital fatalities from haemorrhage were identified. Annual age-standardized mortality rate decreased from 10.97 to 8.18 per 100,000 population (coefficient =  − 0.28, r^2^ = 0.85, *p* =  < 0.001). Trauma was the most common cause (3512, 35.83%) with intentional self-harm (X60–X84), transport accidents (V01–V99) and assault (X85–Y09) being the most common mechanisms of injury. Traumatic fatalities were younger and a larger proportion were male compared to non-traumatic causes (*p* < 0.001). Overall median Charlson Comorbidity Index (Quan) was 0 [0–2] with a lower index noted for traumatic causes (*p* < 0.001). Trauma resulted in a median of 26.1 [3.65–49.22] years of life lost per patient compared to 0 [0–3.65] for non-traumatic causes (*p* < 0.001). Regional variations in mortality rate were observed with lower population density correlating with higher mortality rate (*ρ* =  − 0.64, *p* = 0.002).

**Conclusions:**

Prehospital mortality from haemorrhage decreased between 2012 and 2021. Trauma was the most common cause which resulted in many years of life lost in a population with a low burden of comorbidities. There were considerable regional differences with low population density associated with higher mortality rate from prehospital haemorrhage.

## Introduction

Haemorrhage is a major cause of preventable mortality with causes including trauma, aortic aneurysms, gastrointestinal bleeds, haemoptysis, and pregnancy related haemorrhage. Traumatic haemorrhage has been most well studied and data from modern civilian trauma systems suggest that most fatalities from trauma occur within the first hour due to exsanguination [1, 2].

Emergency care of the bleeding patient has evolved in recent years with the advent of coagulation factor concentrates, antidotes for oral anticoagulants and improved understanding of the role of calcium and fibrinogen. Viscoelastic tests have also enabled goal directed therapy according to the individual needs of each patient. The treatment of massive bleeding typically involves volume replacement with high-ratio blood component or whole blood transfusion typically regarded as superior to intravenous crystalloids. However, there is conflicting evidence regarding prehospital blood transfusion in the prehospital setting [3, 4].

While blood products are lifesaving in some circumstances, they must not be used unnecessarily as they are in limited supply and carry a small risk of complications. Further research is needed to assess the benefit of prehospital blood transfusion in Sweden and the number of prehospital fatalities due to bleeding is a key missing piece of information. This study aims to calculate age-standardized mortality rate due to prehospital haemorrhage on a national and regional level and to describe the fatality’s defining characteristics.

## Methods

The study is a national retrospective cohort study using the Swedish Cause of Death Registry (CDR) and National Patient Registry (NPR) both managed by the Swedish National Board of Health and Welfare. These databases are uniquely complete datasets that can be cross-referenced using unique personal identification numbers. This method has been previously established as a method to identify prehospital deaths in other populations [5–7].

### Data sources

The CDR contains the cause of death for fatalities that occur in Sweden recorded as International Statistical Classification of Diseases, Injuries and Deaths with the tenth edition (ICD-10) in use since 2011. The cause of death is based upon a physician’s report or autopsy. The NPR contains all hospital admissions and out-patient visits to specialist healthcare providers. Population data from the official statistics of Sweden was used to calculate age-standardized mortality rates [8].

### Study population

Patients that met the following criteria were included:

1. A fatality registered in CDR with a date of death between 2012-01-01 and 2021-12-31.
2. The primary cause of death was one of the ICD-10 codes in Appendix 1. For diagnoses resulting from injuries or external causes (S00–T98) all causes were included.
3. The patient was not registered in the NPR:
  - With a date of admission on the same date as the date of death.
  - As currently admitted to a hospital.
4. Has a valid personal identification number.
5. Death occurred in Sweden.

A total of 190 fatalities that matched the first two inclusion criteria were excluded from the study due to an invalid identification number.

### Comorbidities

ICD-10 codes from the five most recent hospital admissions and healthcare visits in the NPR for each fatality were used to calculate Charlson Comorbidity Index (Quan) [9] (CCI) scores.

### Ethical considerations

As the study does not include any identifiable patient data it is exempt from approval by the Swedish Ethical Review Authority. Instead, the Swedish National Board of Health and Welfare reviews the need for ethical approval as part of application for registry data and found that the study was indeed exempt. The dataset was pseudonymized by the National Board of Health and Welfare ensuring that no identifiable patient data reached the authors. We certify that the study was performed in accordance with the ethical standards as laid down in the 1964 Declaration of Helsinki and later amendments or comparable ethical standards.

### Endpoints

The primary outcome was age-standardized mortality rate from prehospital deaths due to haemorrhage per 100,000 inhabitants in Sweden. Secondary outcomes included patient characteristics, comorbidities, causes of death, injury mechanisms and places of residence. Subgroup analyses of the primary and secondary outcomes were performed comparing deaths from trauma with non-traumatic causes.

### Statistical methods

Data was analyzed in R [10] using the tidyverse [11] with *p*-values < 0.05 considered significant. The packages epitools [12] and icd [13] were used to calculate age-standardized mortality rates and comorbidity scores respectively. Age-standardized mortality rates were calculated using direct standardization based on the standard population of Sweden year 2000 [14]. Life years lost were calculated per case by subtracting life-expectancy at birth (according to Statistics Sweden) from age at death with patients less than 1 year old excluded.

## Results

A total of 9801 fatalities from prehospital haemorrhage between 2012 and 2021 were identified and a majority were male (6365, 64.9%). The median age was 71 [55–82] with a range of 0–107 years of age (Table 1). Age-standardized mortality rate ranged from 8.18 to 10.97 fatalities per 100,000 population per year (Table 2) with a significant decline over the study period (coefficient =  − 0.28, r^2^ = 0.85, *p* =  < 0.001).

**Table 1:** Patient characteristics by cause of death

Variable	Overall	Trauma	Gastrointestinal bleeds	Arterial aneurysms and rupture	Other	Haemoptysis	Procedure related	*p*
n (%)	9801	3512 (35.83)	2923 (29.82)	2671 (27.25)	509 (5.19)	162 (1.65)	24 (0.24)	
Male Sex (%)	6365 (64.9)	2708 (77.1)	1662 (56.9)	1607 (60.2)	284 (55.8)	90 (55.6)	14 (58.3)	< 0.001
Age (median [IQR])	71.00 [55, 82]	50.00 [31, 67]	77.00 [66, 87]	77.00 [70, 84]	77.00 [67, 84]	71.00 [65, 79]	77.00 [67.75, 82]	< 0.001
Alcohol related death (%)*	742 (7.6)	140 (4.0)	561 (19.2)	11 (0.4)	27 (5.3)	2 (1.2)	1 (4.2)	< 0.001
Narcotics related death (%)*	58 (0.6)	33 (0.9)	20 (0.7)	4 (0.1)	0 (0.0)	1 (0.6)	0 (0.0)	0.001
*Place of death* (%)								< 0.001
Privat residence	3957 (55.8)	688 (54.7)	1573 (56.6)	1330 (55.3)	252 (52.6)	103 (69.6)	11 (47.8)	
Retirement home	1526 (21.5)	105 (8.4)	1033 (37.2)	276 (11.5)	89 (18.6)	17 (11.5)	6 (26.1)	
Hospital	1608 (22.7)	464 (36.9)	172 (6.2)	800 (33.3)	138 (28.8)	28 (18.9)	6 (26.1)	

ICD-10 diagnoses related to each cause of death are specified in Appendix 1

*IQR* interquartile range

*Alcohol and narcotics related death as classified in the cause of death registry

**Table 2:** Mortality rate from prehospital haemorrhage in Sweden 2012–2021

Year	All causes		Trauma		Other causes	
	n	Mortality rate	n	Mortality rate	n	Mortality rate
2012	1103	10.97	359	3.70	744	7.27
2013	1054	10.37	360	3.64	694	6.73
2014	995	9.63	325	3.27	670	6.36
2015	978	9.35	357	3.55	621	5.81
2016	985	9.28	342	3.36	643	5.92
2017	914	8.44	335	3.27	579	5.18
2018	964	8.88	398	3.87	566	5.01
2019	923	8.36	353	3.35	570	5.01
2020	961	8.57	336	3.15	625	5.42
2021	924	8.18	347	3.28	577	4.89

Mortality rate = annual age standardized mortality rate per 100,000 population

### Causes of death

Non-traumatic causes contributed most to the decrease in mortality rate (coefficient =  − 0.24, r^2^ = 0.81, *p* =  < 0.001) while mortality from traumatic causes remained constant (coefficient =  − 0.04, r^2^ = 0.12, *p* = 0.17). The most common cause of death from prehospital haemorrhage was trauma which accounted for 3512 (35.8%) cases with the most common types of trauma being polytrauma and injuries to the chest (Table 3). Intentional self-half (X60–X84) was the most common mechanism of injury followed by transport accidents (V01–V99) and assault (X85–Y09) (Table 4). The only mechanism of injury which displayed a significant change in mortality rate was assault which increased over the study period (coefficient = 0.03, r^2^ = 0.53, *p* = 0.01). In absolute terms, the number of fatalities due to assault more than doubled from 30 in 2012 to a peak of 67 in 2020. No prehospital fatalities from pregnancy related haemorrhage were seen.

**Table 3:** Traumatic causes of death from prehospital haemorrhage

ICD-10 subchapter	Description	n	% of cases
T00–T07	Injuries involving multiple body regions	1425	46.52
S20–S29	Injuries to the thorax	1279	41.76
S30–S39	Injuries to the abdomen, lower back, lumbar spine and pelvis	134	4.37
S10–S19	Injuries to the neck	107	3.49
S50–S59	Injuries to the elbow and forearm	43	1.40
T08–T14	Injuries to unspecified part of trunk, limb or body region	26	0.85
S80–S89	Injuries to the knee and lower leg	16	0.52
S60–S69	Injuries to the wrist and hand	14	0.46
S70–S79	Injuries to the hip and thigh	12	0.39
S40–S49	Injuries to the shoulder and upper arm	6	0.20
S90–S99	Injuries to the ankle and foot	1	0.03

% of cases = percent of deaths from prehospital haemorrhage in Sweden 2012–2021

**Table 4:** External causes of death from prehospital haemorrhage

ICD-10 subchapter	Description	n	% of all deaths
X60–X84	Intentional self-harm	1415	11.77
V01–V99	Transport accidents	980	1.98
X85–Y09	Assault	443	47.03
W00–W19	Falls	243	2.37
Y10–Y34	Event of undetermined intent	222	6.49
W20–W64	Exposure to inanimate/animate mechanical forces	125	26.04
X58–X59	Accidental exposure to other and unspecified factors	48	0.50
Y35–Y36	Legal intervention and operations of war	13	43.33
Y40–Y84	Complications of medical and surgical care	8	8.79
X40–X49	Accidental poisoning by and exposure to noxious substances	4	0.08
W75–W84	Other accidental threats to breathing	4	0.42
X30–X39	Exposure to forces of nature	2	0.39
Y85–Y89	Sequelae of external causes of morbidity and mortality	2	0.25
W85–W99	Exposure to electric current, radiation and extreme ambient air temperature and pressure	1	2.22
X00–X09	Exposure to smoke, fire and flames	1	0.17

% of all deaths = percent of all deaths from the external cause caused by prehospital haemorrhage in Sweden 2012–2021

### Places of residence

The average annual age-standardized mortality rate per 100,000 population ranged from 6.86 in Region Kronoberg to 11.88 in Region Norrbotten (Table 5). Lower population density was correlated with higher average overall mortality rates (*ρ* =  − -0.64*, p* = 0.002).

**Table 5:** Average standardized mortality rate by place of residence

Region	Population density	All causes	Trauma	Non-traumatic
		Mortality rate	Mortality rate	Mortality rate
Norrbotten	2.59	11.88	4.20	7.69
Örebro	34.80	11.73	3.70	8.03
Gävleborg	15.66	11.70	4.62	7.09
Västernorrland	11.32	11.12	3.13	7.98
Gotland	18.68	10.67	4.11	6.56
Östergötland	42.93	10.57	3.33	7.24
Västerbotten	4.88	10.18	3.28	6.90
Jämtland	2.64	10.16	2.88	7.28
Kalmar	21.58	9.90	4.20	5.70
Södermanland	47.57	9.88	3.78	6.10
Dalarna	10.12	9.61	3.87	5.73
Värmland	15.89	9.44	3.89	5.55
Stockholm	350.06	8.98	3.58	5.40
Västmanland	52.47	8.66	3.25	5.40
Jönköping	33.90	8.66	2.93	5.73
Skåne	121.56	8.54	2.99	5.54
Blekinge	53.58	8.51	2.68	5.83
Uppsala	44.74	8.34	3.28	5.06
Västra Götaland	70.47	7.89	3.40	4.50
Halland	59.36	7.39	2.43	4.96
Kronoberg	23.16	6.86	2.45	4.41

Mortality rate = average annual age-standardized mortality rate per 100,000 population

Population density = number of inhabitants per square kilometer

### Comorbidities and years of life lost

The study population had a low burden of comorbidities with a median overall CCI of 0 [0–2] and the CCI was lower in patients deceased due to trauma compared to non-traumatic causes (*p* < 0.001). All types of Charlson comorbidities were more prevalent in the non-traumatic causes group except HIV/AIDS and mild liver disease (Table 6). A total of 97,771 years of life were lost due to trauma over the study period which was more than all other causes combined (arterial aneurysm and rupture = 8219, gastrointestinal bleeds = 13,939, haemoptysis = 1048, other = 2112, procedure related = 140). Trauma resulted in a median of 26.1 [3.65–49.22] years of life lost per patient compared to 0 [0–3.65] for non-traumatic causes (*p* < 0.001). A total of 863 (8.81%) patients were on anticoagulants (Z92.1) which were more prevalent in the non-traumatic causes group (152, 11.30%) compared to traumatic causes (711, 4.32%) (*p* < 0.001).

**Table 6:** Charlson comorbidities (Quan)

Comorbidity	Overall	Trauma	Non-traumatic	*p*
Myocardial infarction	7.10%	3.43%	9.09%	< 0.001
Congestive heart failure	10.39%	4.40%	13.65%	< 0.001
Peripheral vascular disease	15.23%	1.24%	22.83%	< 0.001
Cerebrovascular disease	9.70%	4.54%	12.51%	< 0.001
Dementia	6.73%	2.29%	9.15%	< 0.001
Chronic pulmonary disease	9.82%	4.77%	12.57%	< 0.001
Connective tissue disease	3.20%	1.24%	4.26%	< 0.001
Peptic ulcer disease	3.53%	1.04%	4.88%	< 0.001
Mild liver disease	3.05%	2.89%	3.13%	0.596
Diabetes without complications	8.10%	4.40%	10.12%	< 0.001
Diabetes with complications	2.04%	1.28%	2.45%	< 0.001
Hemi- or paraplegia	2.03%	0.94%	2.62%	< 0.001
Renal disease	5.30%	1.98%	7.10%	< 0.001
Cancer	8.68%	3.67%	11.42%	< 0.001
Severe liver disease	1.99%	0.40%	2.85%	< 0.001
Metastatic solid tumor	4.31%	1.04%	6.09%	< 0.001
HIV/AIDS	0.04%	0.07%	0.02%	0.285
CCI (median [IQR])	0 [0–2]	0 [0–0]	1 [0–2]	< 0.001

*CCI* Charlson comorbidity index (Quan), *IQR* interquartile range

*P*-values refer to differences between traumatic and non-traumatic causes using Fishers exact test for all comorbidities and Mann–Whitney U test for CCI

## Discussion

This study represents the first time that all prehospital fatalities from haemorrhage on a national level have been studied over a consecutive time period. A key finding was the significant decline in prehospital mortality rate from haemorrhage which was mostly attributed to non-traumatic causes.

### Patient demographics

Recent data from a Swedish trauma center [15] found that median age in bleeding trauma patients was 39 years and a study from Texas, USA [2] found similar results with a median age of 35.4 in haemorrhagic deaths. Prehospital fatalities from traumatic haemorrhage had a higher median age of 50 which suggests that age may be a risk factor for rapid demise before arrival at hospital in setting of traumatic haemorrhage. Males were overrepresented in the present study which is consistent with the incidence of major bleeding in trauma [15].

### Causes of death

Prehospital haemorrhage accounted for approximately 1% of all deaths, 11.77% of suicide and 47.03% of fatalities from assault in Sweden over the study period [16]. Overall national age-standardized mortality rate from all causes decreased over the study period whereas mortality from external causes (V01–Y98) did not change significantly [16]. The changes in mortality rate from prehospital haemorrhage followed a similar pattern. The advent of direct oral anticoagulants and screening for aortic aneurysms are two possible contributing factors which have both contributed to decreased mortality from bleeding [17, 18].

### Places of residence

Regional differences in age-standardized mortality rate were considerable with Sweden’s most northerly region of Norrbotten exhibiting 1.7 times higher mortality rate compared to Kronoberg in the south. Higher population density was negatively correlated with mortality rate which raises the question if there are regional differences in prehospital care that influence prehospital mortality from haemorrhage.

Prehospital blood transfusion has been shown to be especially beneficial when transport times exceed 20 min [19] and a recent study found that median transport time was 47 min for severely bleeding trauma patients in Sweden’s largest city of Stockholm [15]. Transport times appear to be an important target of future investigations to determine which regions may have the greatest benefit of prehospital blood transfusion programs.

### Comorbidities and years of life lost

The study illustrates that previously healthy individuals are susceptible to massive haemorrhage especially when caused by trauma. Interestingly, traumatic deaths from prehospital haemorrhage were associated with a lower burden of comorbidities which contrasts with previous studies which have found CCI to predict mortality after trauma [20]. The prevalence of atrial fibrillation in Sweden is approximately 3% [21] which suggests that patients on anticoagulants are overrepresented in the study population. Further research is needed to determine if anticoagulants are associated with an increased risk of prehospital massive haemorrhage.

### Limitations

Classification of a fatality as caused by haemorrhage was done based on ICD-10 codes on a registry level. The included ICD-10 codes were chosen based on the reasoning that bleeding is the most probable consequence of an injury or disease for it to be fatal outside of hospital. We believe this is the best possible representation of haemorrhagic deaths at the national level. However, there is a small degree of uncertainty regarding the degree and type of bleeding particularly in severely traumatized patients who often have multiple injuries.

Retrospective studies have inherent limitations including the reliability of registry data which the present study mitigates by using reliable government registries. For example, the CDR is a complete dataset for Swedish residents and a cause of death is available for > 99% of fatalities [14]. Data quality is regarded as high since reporting is mandatory, related to remuneration and data is aggregated from electronic medical and tax records [22]. Additionally, cause of death was based on autopsy in a majority of cases (6,608, 67.42%) which results in improved reliability compared to the general population where autopsy is less common [23].

Cross-referencing the CDR and NPR requires a valid personal identification number meaning that non-residents were excluded. Over the study period there were 10–30 fatalities per year with an invalid identification number that matched the diagnosis criteria. However, these fatalities could have occurred anywhere meaning the number of prehospital cases are much fewer and thus unlikely to impact the study’s results.

The study classifies a fatality as prehospital when there is no hospital admission on the date of death. However, the place of death was notably still recorded as “hospital” on the death certificates of 1,608 (22.7%) patients. These patients were likely deemed possible survivors by first responders yet were impossible to resuscitate on arrival. Changes to emergency treatment of massive haemorrhage would potentially have a large impact on this subgroup making it an especially interesting target of future investigations.

## Conclusions

There was a significant decrease in prehospital mortality from haemorrhage between 2012 and 2021 and non-traumatic causes contributed most to the decline. The most common cause of death from prehospital haemorrhage was trauma which resulted in many years of life lost in a patient population with a low burden of comorbidities. Large regional differences in mortality were noted with higher mortality rate correlating with lower population density. The study provides novel insight into the epidemiology of prehospital fatalities from haemorrhage on a national level which may help guide prehospital transfusion programs and promote the responsible use of blood products in Sweden and similar healthcare systems.

## Data Availability

The data that support the findings of this study are available from the Swedish Board of Health and Welfare pending an approved application. Certain data on a group level is publicly available at: https://sdb.socialstyrelsen.se/if_dor/val.aspx (cited as Socialstyrelsen. Dödsorsaker [Socialstyrelsens statistikdatabas]. Stockholm; 2024).

## Appendix 1

**Table:**

ICD-code	
*Arterial aneurysms and rupture*	
I71	Aortic aneurysm
I72	Other aneurysms
I77.2	Arterial rupture
I79	Aneurysm in aorta
*Gastrointestinal bleeds*	
I85	Oesophageal varices
K22	Other diseases of oesophagus
K25	Gastric ulcer
K26	Duodenal ulcer
K27	Peptic ulcer, site unspecified
K28	Gastrojejunal ulcer
K62.5	Haemorrhage of anus and rectum
K66.1	Haemoperitoneum
K92	Other diseases of digestive system
*Pregnancy related haemorrhage*	
O20	Haemorrhage in early pregnancy
O44.1	Placenta praevia with haemorrhage
O46	Antepartum haemorrhage, not elsewhere classified
O72	Postpartum haemorrhage
*Haemoptysis*	
R04	Haemorrhage from respiratory passages
*Other causes of haemorrhage*	
D62	Acute posthaemorrhagic anaemia
D69.9	Haemorrhagic condition, unspecified
R57.1	Hypovolemic shock
R58	Haemorrhage, not elsewhere classified
*Procedure related haemorrhage*	
T81.0	Haemorrhage and haematoma complicating a procedure, not elsewhere classified
*Trauma*	
S11	Open wound of neck
S18	Traumatic amputation at neck level
S19	Other and unspecified injuries of neck
S21	Open wound of thorax
S25	Injury of blood vessels of thorax
S26	Injury of heart
S27	Injury of other and unspecified intrathoracic organs
S28	Crushing injury of thorax and traumatic amputation of part of thorax
S29	Other and unspecified injuries of thorax
S31	Open wound of abdomen, lower back and pelvis
S35	Injury of blood vessels at abdomen, lower back and pelvis level
S36	Injury of intra-abdominal organs
S37	Injury of urinary and pelvic organs
S38	Crushing injury and traumatic amputation of part of abdomen, lower back and pelvis
S39	Other and unspecified injuries of abdomen, lower back and pelvis
S41	Open wound of shoulder and upper arm
S45	Fracture of shoulder and upper arm
S47	Crushing injury of shoulder and upper arm
S48	Traumatic amputation of shoulder and upper arm
S49	Other and unspecified injuries of shoulder and upper arm
S51	Open wound of forearm
S55	Injury of blood vessels at forearm level
S57	Crushing injury of forearm
S58	Traumatic amputation of forearm
S59	Other and unspecified injuries of forearm
S61	Open wound of wrist and hand
S65	Injury of blood vessels at wrist and hand level
S67	Crushing injury of wrist and hand
S68	Traumatic amputation of wrist and hand
S69	Other and unspecified injuries of wrist and hand
S71	Open wound of hip and thigh
S75	Injury of blood vessels at hip and thigh level
S77	Crushing injury of hip and thigh
S78	Traumatic amputation of hip and thigh
S79	Other and unspecified injuries of hip and thigh
S81	Open wound of lower leg
S85	Injury of blood vessels at lower leg level
S87	Crushing injury of lower leg
S88	Traumatic amputation of lower leg
S89	Other and unspecified injuries of lower leg
S91	Open wound of ankle and foot
S95	Injury of blood vessels at ankle and foot level
S97	Crushing injury of ankle and foot
S98	Traumatic amputation of ankle and foot
S99	Other and unspecified injuries of ankle and foot
T00–07	Injuries involving multiple body regions
T09–14	Injuries to unspecified parts of trunk, limb or body region
T79.2	Traumatic secondary and recurrent haemorrhage
T79.4	Traumatic shock

## Abbreviations

CDR: Cause of death registry
NPR: National patient registry
ICD-10: International statistical classification of diseases, injuries and deaths—10th edition
CCI: Charlson comorbidity index (Quan)
